# Smart Contact Lenses: Disease Monitoring and Treatment

**DOI:** 10.34133/research.0611

**Published:** 2025-02-10

**Authors:** Meidie Pan, Zhuohao Zhang, Luoran Shang

**Affiliations:** ^1^ Department of Ophthalmology, Eye and ENT Hospital of Fudan University, Shanghai 200032, China.; ^2^ Institutes of Biomedical Sciences, Fudan University, Shanghai 200032, China.

## Abstract

Smart contact lenses (SCLs), an innovative evolution of conventional contact lenses, have recently attracted increasing attention for their substantial potential for use in the healthcare field. With advancements in materials science and medical technology, SCLs have integrated electronic information technology with biomedical engineering to enable the incorporation of various medical functionalities. Recent developments have focused on applying SCLs to provide intelligent, efficient, and personalized healthcare solutions in the surveillance, diagnosis, and treatment of chronic ocular surface inflammation, glaucoma, and diabetes complications.

Contact lenses have long been favored for their use in visual correction, as they offer a convenient and comfortable alternative to traditional eyeglasses. These lenses have become widely adopted as the preferred choice by many individuals, following the establishment of a solid reputation for their safety and practicability. Further, recent advances in materials science and biomedical technologies have catalyzed the evolution of contact lenses into smart contact lenses (SCLs), which integrate electronic information technology with biomedicine to achieve multiple medical functionalities [1].

SCLs can correct vision while playing a vital role in healthcare, including disease surveillance, diagnosis, and treatment [2]. By maintaining continuous contact with tears, which are an integral part of bodily fluid, SCLs are ideally suited for detecting a variety of biological signals from the eyes. The chemical composition of tears, which is closely correlated with biomarkers in the blood due to the blood–tear barrier, further supports their use in assessing systemic biomarkers [3]. Moreover, the incorporation of near-field communication (NFC) chips into certain SCLs can enable real-time visualization of health data on smartphones, aligning with the growing demand for precise, comprehensive, and personalized healthcare [1]. Consequently, substantial efforts are currently being directed toward the development of optimized detection strategies for various biomarkers, thereby expanding the potential applications of SCLs.

Recently, substantial advances have been made in the application of SCLs, including their use in the monitoring of blood biochemical indicators and intraocular pressure (IOP), as well as in the diagnosis and treatment of various diseases [3–7]. Notably, in March 2021, Jang et al. [4] introduced an SCL for the diagnosis and treatment of chronic ocular surface inflammation (OSI) diseases. OSI diseases, encompassing conditions such as dry eye syndrome and meibomian gland dysfunction, are becoming increasingly prevalent. However, their diagnosis remains challenging because of the presence of atypical clinical signs and symptoms in some patients, which can lead to false negatives. To address this, Jang et al. took advantage of the highly specific antigen–antibody recognition mechanism and created a graphene field-effect transistor biosensor embedded within a contact lens (Figure). This biosensor, functionalized with a matrix metalloproteinase-9 (MMP-9)-specific antibody, can detect the concentration of MMP-9, a key biomarker of OSI, in tears by altering its electrical signal. This sensor demonstrated exceptional responsiveness across a range of MMP-9 concentrations from 1 to 500 ng ml^−1^, which covers that of the diagnostic criteria for OSI. Such a sensor was integrated with other electronic elements as well as an antenna and an NFC chip, allowing the construction of an SCL for personalized and convenient monitoring of OSI. Moreover, the proposed SCL maintained its stability even after mechanical deformation, making it adaptable to the flexible nature of soft contact lenses. When complemented with heat patches designed for eyelid attachment, this integrated device offers an individualized approach for the diagnosis and treatment of chronic OSI, enabling continuous and noninvasive health management. Both the SCL and the patches were tested in rabbits and human beings to verify their safety and efficacy.

**Figure.**
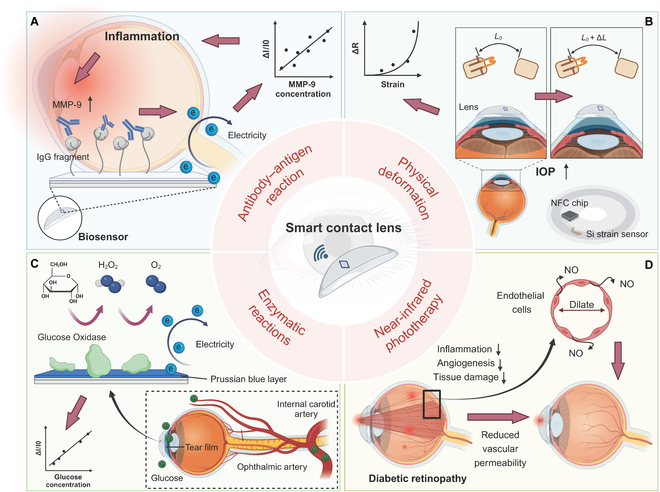
Schematic illustration of the working mechanisms of typical smart contact lenses (SCLs) for disease monitoring and treatment. (A) Detection of ocular surface inflammation through antigen–antibody responses. (B) Monitoring of intraocular pressure (IOP) via physical deformation of the lens. (C) Measurement of glucose levels in tears using an electrochemical method. (D) Utilization of far-red/near-infrared light in the treatment of diabetic retinopathy. MMP-9, matrix metalloproteinase-9; IgG, immunoglobulin G; NFC, near-field communication.

Building on their success in addressing chronic OSI, Kim et al. [5] extended their innovative approach to tackle glaucoma, a leading cause of irreversible blindness, by monitoring IOP and had success in rabbits and humans. Given the critical role of IOP as a modifiable risk factor for glaucoma progression, the team engineered an SCL with a strain sensor and specially patterned soft and rigid regions to concentrate the tiny deformations caused by changes in IOP (Figure). Such design allows the device to detect minute changes with exceptional sensitivity without the use of additional data amplifier or noise filter, achieving a remarkable resolution of 0.014 mmHg (surpassing that of existing commercial devices). In addition, this technology enables wireless data transmission to smartphones using NFC technology, and the integration of a software facilitated self-calibration of the IOP readout, greatly enhancing strain measurement accuracy. Notably, this SCL demonstrated outstanding softness owing to the freestanding liquid metal EGaIn interconnections as well as stretchable antenna composed of hybrid silver nanofibers and silver nanowires. Compared to the gold-standard tonometry, the SCL presents a simple yet effective solution for real-time IOP monitoring, which is crucial for the early detection and management of glaucoma.

Following their achievements in developing SCLs for localized eye conditions, Park et al.’s lab turned their attention to systemic health monitoring. They constructed SCLs with an electrochemical glucose sensor for monitoring tear glucose (TG) as an alternative of blood glucose (BG) [6]. In this system, glucose oxidase immobilized on the working electrode of the electrochemical sensor reacts with glucose in tears to produce gluconolactone and H_2_O_2_. The resulting H_2_O_2_ is subsequently reduced by Prussian blue, and the glucose concentration can be determined by measuring the change of current (Figure). Impressively, the sensor could reach a limit of detection for glucose of 0.02 mM, confirming its capability to monitor TG levels. To further validate the correlation between TG and BG, oral glucose tolerance tests in normal rabbits and intravenous glucose tolerance tests in both normal and diabetic rabbits were carried out. An in-depth correlation analysis was conducted, confirming that this approach allowed the real-time and dynamic measurement of glucose concentrations, thereby providing valuable insights into the TG–BG relationship. Notably, the authors also introduced the concept of “personalized lag time” to account for individual variations in glucose metabolism, demonstrating that this metric could be calibrated for each user to achieve precise BG monitoring. Their study, which also included rigorous testing in beagles and human pilot study, meticulously considered the differences in glucose metabolism caused by various dietary habits and underscored the potential of SCLs to deliver accurate health guidance to wearers and offer critical information to medical professionals.

In addition to diagnostics, the application of SCLs has been extended to therapeutic interventions. Previous studies have proposed the use of SCLs to control drug delivery or integrate phototherapeutic agents to treat ocular diseases [7,8]. A team led by Lee et al. [7] developed a near-infrared-light-therapy-based SCL to treat diabetic retinopathy (Figure). This SCL integrated a wireless light-emitting diode capable of emitting light in the range of 630 to 1,000 nm, and a wavelength of ≈670 nm was selected specifically for the treatment of diabetic retinopathy. The intensity of the emitted light was remotely adjusted to ensure safety and efficacy throughout the treatment process. Subsequent monitoring of the molecular biomarkers and the main clinical symptoms from rabbits of the disease demonstrated that the use of the light-emitting diode SCLs for 15 min, 3 times a week, with 120-μW light exposure over an 8-week period effectively improved diabetic retinopathy. Moreover, measurement of the retina thickness revealed that this phototherapeutic approach mitigated the retinal nerve damage, which may particularly benefit patients at the early stage. The mechanism underlying this effect likely involves the stimulation of endothelial cells to produce nitric oxide, which dilates blood vessels and alleviates hypoxia, thereby reducing pathological neovascularization and preserving the normal structure and function of the retina. Compared with conventional vitreoretinal injections or surgical interventions, this noninvasive treatment method can greatly enhance patient compliance, thereby offering a more accessible and comfortable therapeutic option.

SCLs have garnered considerable attention as precise, noninvasive, real-time wearable medical devices. Despite these advancements, SCL technology still faces several challenges. For example, improving SCLs’ stability is a critical task for commercialization purposes. SCLs must be sufficiently durable to withstand disinfection or any type of harsh environment while maintaining functionality over extended periods [9]. Also, it is necessary to ensure that both SCLs and their degradation products maintain good biocompatibility without irritations or harm to the ocular surface microenvironment and corneal epithelium. At the same time, miniaturization, power supply, manufacturing, regulatory approvals, and market uncertainties are all issues that SCLs need to face as they moves toward commercialization [10].

Despite these challenges, with advancements in materials and engineering technologies, the capabilities of SCLs have expanded continuously, for both disease monitoring and treatment. The development of some advanced materials has greatly improved the oxygen permeability of contact lenses and can highly balance oxygen permeability, water content, and mechanical properties, making it possible to wear contact lenses for a long time and ameliorate clinical complications such as superficial epithelial lesions of the cornea and elevated tear mucin globule content [11,12]. These advances provide a platform technology for the development of SCLs. In addition, the application of SCLs has the potential to broaden beyond the eye. Given that the retina shares an ectodermal origin with the central nervous system and that some studies suggest its involvement in neurodegenerative diseases, SCLs may serve as noninvasive tools for central nervous system assessments [13]. Furthermore, one notable area for growth is the integration of multifunctional capabilities. The convergence of advanced sensor technologies and rapidly evolving medical artificial intelligence facilitates real-time physiological monitoring and rapid therapeutic interventions within both wearable devices and broader healthcare systems [14,15]. The integration of sophisticated algorithms and artificial-intelligence-driven analytical frameworks enables comprehensive data analysis, thereby facilitating life cycle management and enhancing patient quality of life.

Overall, we believe that advancements in electronic information technology, materials science, and biomedicine will propel SCLs to play a more important role in enhancing overall healthcare in the foreseeable future.
